# A Community-Based Short Message Service Intervention to Improve Mothers’ Feeding Practices for Obesity Prevention: Quasi-Experimental Study

**DOI:** 10.2196/13828

**Published:** 2019-06-03

**Authors:** Hong Jiang, Mu Li, Li Ming Wen, Louise Baur, Gengsheng He, Xiaoying Ma, Xu Qian

**Affiliations:** 1 School of Public Health Global Health Institute Fudan University Shanghai China; 2 Key Lab of Health Technology Assessment, National Health Commission of the People's Republic of China Fudan University Shanghai China; 3 School of Public Health University of Sydney Sydney Australia; 4 China Studies Centre University of Sydney Sydney Australia; 5 Health Promotion Unit Sydney Local Health District Sydney Australia; 6 Discipline of Child & Adolescent Health University of Sydney Sydney Australia

**Keywords:** short message service, child development, body mass index, BMI, childhood obesity

## Abstract

**Background:**

The prevalence of childhood obesity is increasing in China, and the effect of mobile phone short message service (SMS) interventions to prevent early childhood obesity needs to be evaluated.

**Objective:**

The objective of this study was to assess the effect of an SMS intervention on the prevention of obesity in young children.

**Methods:**

A quasi-experimental design SMS intervention was carried out in 4 community health centers (CHCs) in Shanghai, China. A total of 2 CHCs were assigned to the intervention group, and 2 CHCs were assigned to the control group. Mothers in the intervention group received weekly SMS messages on breastfeeding and infant feeding from the third trimester to 12 months postpartum. The primary outcomes were children’s body mass index (BMI), BMI z-score, and weight-for-length z-score at 12 and 24 months. Factors associated with higher BMI and weight-for-length z-score at 24 months were also assessed.

**Results:**

A total of 582 expectant mothers were recruited at the beginning of the third gestational trimester. 477 (82.0%) and 467 (80.2%) mothers and their children were followed up to 12 and 24 months postpartum, respectively. There were no significant differences in children’s BMI, BMI z-score, and weight-for-length z-score at 12 and 24 months between the 2 groups. Factors associated with higher BMI, BMI z-score, and weight-for-length z-score at 24 months included higher birth weight, introduction of solid foods before 4 months, and taking a bottle to bed at 12 months.

**Conclusions:**

The SMS intervention did not show a significant effect on children’s BMI, BMI z-score, or weight-for-length z-score at 12 and 24 months. Introduction of solid foods before 4 months and taking a bottle to bed at 12 months were significantly and positively correlated with a higher BMI, BMI z-score, and weight-for-length z-score at 24 months. Further studies with more rigorous design are needed to evaluate the effect of SMS interventions on preventing early childhood obesity.

## Introduction

### Background

Childhood obesity is a major public health concern globally. In China, the prevalence of obesity in children and adolescents aged 0 to 18 years increased from 0.4% in 1985 to 7.5% in 2010, a more than 18-fold increase in 25 years [1]. Early childhood, particularly the prenatal period, is a critical period to prevent obesity in later life [2,3]. There has been an increased interest in research on early childhood obesity interventions in recent years, including home- or caregiver-based interventions [4,5], interactive training modules for parents, and environmental change to promote healthy eating and active playing [6,7]. Such interventions have reportedly led to improvements in infant feeding practices (eg, increased duration of breastfeeding and a decrease in soft drink consumption) and a reduction in TV viewing while eating [8,9]. One study has also shown a reduction in body mass index (BMI) at age 24 months [8].

Mobile health (mHealth) refers to delivering health care and health promotion through mobile devices [10]. Mobile phone short message service (SMS) can deliver health care inexpensively through text messages wherever the person is located [11]. SMS text messaging has attracted global attention for its ability to enhance health care services [12,13]. Reported SMS text messaging in maternal and child health care are extensive, such as promoting prenatal care utilization, promoting exclusive breastfeeding, and improving the training of maternal and child health care providers [10,11,14]. The effects of these interventions, however, are underevaluated.

### Aims

We implemented an SMS intervention to first-time mothers in Shanghai, China. The overall aims of the study were to promote healthy infant feeding practices to new mothers and to examine whether the intervention is effective in preventing early onset of childhood obesity of their children. We have previously reported significant improvements in median exclusive breastfeeding (EBF) duration and rate of EBF at 6 months among the mothers receiving the intervention [15]. In this paper, we report the effect of this SMS intervention on the BMI, BMI z-score, and weight-for-length z-score of the children at 12 and 24 months. We also present factors associated with higher BMI, BMI z-score, and weight-for-length z-score at 24 months.

## Methods

### Study Design

This SMS text messaging intervention, using a quasi-experimental design, was conducted in Shanghai, China. A total of 4 community health centers (CHCs) were purposively selected from 2 administrative districts; 2 CHCs were assigned as the intervention group, and the other 2 were assigned to the control group. The intervention group received a weekly mobile phone SMS on appropriate infant feeding practices, plus routine child health care provided by CHCs. Participating mothers in the control group only received the routine child health care services. The intervention was carried out between December 2010 and October 2012. The 24-month anthropometric data collection was completed in October 2013. Written informed consent was obtained from each participant. Ethics approval was obtained from the Institutional Review Board of the School of Public Health, Fudan University, Shanghai, China, and the Human Research Ethics Committee of the University of Sydney, Sydney, Australia [15].

### Primary and Secondary Outcomes

The primary outcomes of this intervention were child’s BMI, BMI z-score, and weight-for-length z-score at 12 and 24 months. Length and weight data at 12 and 24 months were analyzed. Secondary outcomes were the proportion of infants introduced to solid foods before 4 months and taking a bottle to bed at 12 months; their associations with BMI at 24 months were also investigated.

### Participants and Recruitment

The inclusion criteria and recruitment processes have been reported previously [15]. Essentially, eligible expectant mothers, owning a mobile phone, from the 4 communities were recruited to the study when they attended their first antenatal checkup in the CHCs after giving informed consent. They were asked to complete a baseline self-administered questionnaire, including questions on their initial awareness of the World Health Organization (WHO) breastfeeding guidelines [16]. CHC staff reviewed the participants’ eligibility again at the beginning of the third trimester before the commencement of the SMS intervention.

### Sample Size

To detect a difference in BMI of 0.4 units between the intervention and control groups at 24 months at the significance level of *P*<.05 with 80% power, a sample size of 446 (223 per arm) was needed. The estimation was made on the assumption of SDs of 1.4 and 1.6 for BMI at 24 months based on a published survey. Given an estimated loss to follow-up rate of 20% at the end of 24 months, 558 expectant mothers were required for the study.

### Short Message Service Intervention

One weekly text message was sent to the participants in the intervention group from a computer-based platform from 28 weeks gestation to 12 months postpartum [15]. The total duration of the intervention was thus 66 weeks. Each text message contained approximately 180 to 210 characters. The messages were developed from the WHO breastfeeding guidelines and infant and young child feeding recommendations, in consultation with child health care experts and informed by a formative study [17]. The messages covered different stages of infant growth and development, providing anticipatory knowledge and guidance for appropriate infant feeding practices [15]. In addition, messages were sent periodically inquiring about the breastfeeding status, the time to return to work, or the timing of introducing semisolids or solids, so that appropriate messages could be sent in response to each woman’s feeding situation. The control group received routine maternal and child health care in the CHCs. The control group received maternal and child health care routinely provided by the CHCs, which included anthropometric measurements of length and weight periodically, responses to child feeding enquires, and assessment of the child development.

### Data Collection and Main Measurements

Child health care doctors from the 4 CHCs were trained for standard measurement and data collection procedures at the beginning of the study. The data on EBF, breastfeeding, and timing of introducing solids were collected and recorded by doctors via face-to-face interviews [15]. Child weight (to the nearest 0.1 kg) and length (to the nearest 0.1 cm) at 12 and 24 months were measured by the doctors using established methods and recorded using a standard protocol as part of a routine child health checkup [18]. Information on breastfeeding, timing of introducing semisolids and solids, and anthropometric measurement were extracted from the children’s health records. Children’s BMI at 12 and 24 months was calculated as weight in kg/(length in m)^2^, and BMI z-score and weight-for-length z-score were calculated using the lambda-mu-sigma method based on the WHO Child Growth Standards [19]. At 12 months postpartum, a face-to-face interview was held with mothers by CHC staff in each CHC using a questionnaire adapted and translated from the Healthy Beginnings Trial [20]. Information on care givers’ feeding practices, such as drinking from a cup, giving a bottle at bedtime, and using food for reward, was collected [15].

### Statistical Analysis

Data quality control was performed before data analysis. The data were excluded if children’s weight gain or length was less than 0.5 kg or 3 cm between birth weight and 12 months and between 12 and 24 months without any clinical indications.

Statistical analyses were made using the SPSS for Windows, version 17.0. Continuous variables were compared using independent samples *t* test, proportions using the Pearson chi-squared test, and trends in proportions using Mantel-Haenszel chi-squared tests. Statistical significance level was set at *P*<.05.

Awareness of WHO breastfeeding guidelines was assessed with 6 questions in the baseline questionnaire. Each correct answer was scored 1 and an incorrect scored 0. The total scores ranged from 0 to 6, categorized into *high* or *low* score groups according to the median score.

BMI, BMI z-score, length, weight, and weight-for-length z-score at 12 and 24 months were analyzed as continuous variables. The effect of intervention on BMI, BMI z-score and weight-for-length z-scores was determined by analysis of variance (ANOVA) after controlling for baby’s birth weight; mothers’ age, education level, preconception maternal BMI; household registration status, whether living in rental accommodation; and baseline awareness of WHO breastfeeding guidelines. The effect of the intervention on the child’s BMI and BMI z-score was analyzed in the same way, with additional adjustment for baby’s sex. As children might not be measured exactly when turning 12 or 24 months, further adjustment was made for the anthropometric data collection by exact child age (months to 2 decimal point). Similarly, the analysis of length and weight were further controlled for mother’s preconception height and weight, respectively.

Multivariate linear regression was applied to analyze the factors associated with BMI, BMI z-score, and weight-for-length z-score at 24 months. Factors in the model included baby’s birth weight; mothers’ age and education level, awareness of the WHO breastfeeding guidelines at baseline, preconception BMI, EBF status at 6 months, breastfeeding duration (weeks), and introduction of solid foods before 4 months; care givers’ feeding practices at 12 months, such as drinking from a cup, taking a bottle to bed, and using food for reward; and whether the family had Shanghai household registration and whether the family was living in a rental accommodation.

## Results

### Recruitment and Follow-Up

**Figure 1:**
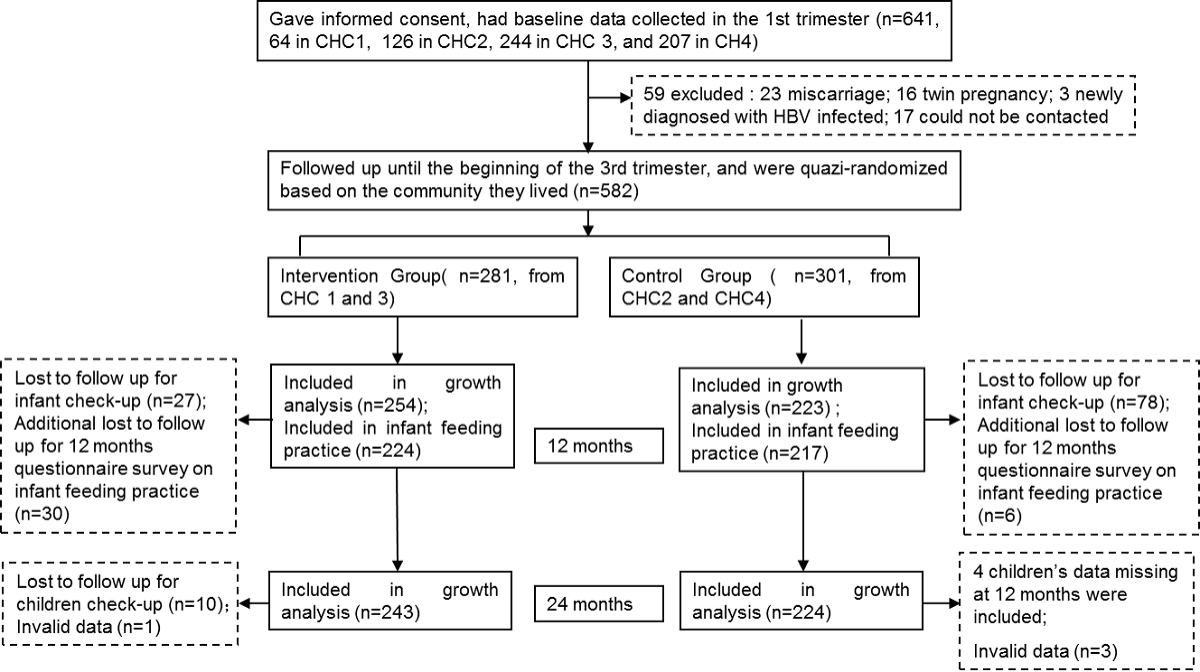
Participant recruitment and retention. CHC: community health center; BMI: body mass index; HBV:hepatitis b virus.

Of the 641 expectant mothers recruited from the 4 CHCs during their first visit, 582 were eligible at the beginning of the third trimester, 281 in the intervention group and 301 in the control group. There was no significant difference on main characteristics between women recruited in the first trimester and eligible in the third trimester. At 12 months, 82.0% (478/582) of the children’s weight and length measurements were obtained, and 75.7% (441/582) of the mothers completed the questionnaire survey. At 24 months, 80.2% (467/582) of the children’s anthropometric data were collected (Figure 1).

### Participants’ Main Characteristics

Multimedia Appendix 1 shows the main characteristics of participants at baseline and at 12 and 24 months. At baseline, the mean age of the mothers was 28 years (range: 20-39 years). 75.6% (440/582) of the mothers did not hold Shanghai household registration, and 86.4% (503/582) had tertiary education. Nearly one-half of mothers (44.7%, 260/582) had a low awareness of WHO breastfeeding guidelines. Mothers who were lost to follow-up at 12 months were more likely to be living in a rental accommodation (*P*<.001) and had a higher awareness score of WHO breastfeeding guidelines (*P*=.02). Similarly, mothers who were lost to follow-up at 24 months were more likely to be living in a rental accommodation (*P*<.001) and had a non-Shanghai household registration status (*P*=.03). At 12 months, the average BMI of the children was 17.04 kg/m^2^ (SE 0.06) ranging from 13.65 to 21.04, and BMI z-score was 0.46 (SE 0.04), with the range of −1.96 to 2.85. At 24 months, the average BMI of the children was 15.92 kg/m^2^ (SE 0.05) ranging from 12.62 to 20.22, and the mean BMI z-score 0.05 (SE 0.04) ranging from −2.69 to 2.83.

### Effect of the Intervention on Body Mass Index, Body Mass Index Z-Score, and Weight-for-Length Z-Score

By ANOVA, there was no statistically significant difference between the intervention and control groups in BMI (mean 17.04 kg/m^2^, SE 0.08 vs mean 17.04 kg/m^2^, SE 0.08), BMI z-score (mean 0.47, SE 0.05 vs mean 0.46, SE 0.06), or weight-for-length z-score (mean 0.44, SE 0.05 vs mean 0.44, SE 0.05) at 12 months. Similar results were found at 24 months for BMI (mean 15.93 kg/m^2^, SE 0.07 vs mean 15.90 kg/m^2^, SE 0.08), BMI z-score (mean 0.06, SE 0.06 vs mean 0.04, SE 0.06), or weight-for-length z-score (mean 0.44, SE 0.05 vs mean 0.23, SE 0.06), respectively (Multimedia Appendix 2). No difference was found in length and weight between children in intervention and control groups at 12 months and 24 months.

### Factors Associated With Body Mass Index and Body Mass Index Z-Score at 24 Months

Table 1 shows the results of multiple linear regression analysis of factors associated with BMI, BMI z-score, and weight-for-length z-score at 24 months. The factors significantly and positively correlated with BMI, BMI z-score, and weight-for-length z-score at 24 months included higher birthweight (all *P*<.001), introduction of solid foods before 4 months (*P*=.003, *P*=.004, and *P*=.003, respectively), and taking a bottle to bed at 12 months (*P*=.048, *P*=.044, and *P*=.036, respectively).

**Table 1:** Factors associated with body mass index (BMI) and BMI z-score at 24 months (n=359).

Variables		Beta^a^ (95% CI)	*P* value^a^
**BMI^b^** **at 24 months**			
	Household registration	−.144 (−.416 to .129)	.30
	Rental accommodation	−.162 (−.495 to .172)	.34
	Awareness of WHO^c^ breastfeeding guidelines at baseline	.008 (−.222 to .248)	.95
	Maternal pre-pregnancy BMI	.020 (−.027 to .067)	.40
	Maternal education	.013 (−.265 to .291)	.93
	Birthweight	.612 (.343 to .881)	<.001
	Baby’s sex	.113 (−.121 to .347)	.34
	Exact child’s age at follow-up (month)	−.139 (−.378 to .099)	.25
	Maternal age at recruitment	−.045 (−.252 to .163)	.67
	EBF^d^ at 6 months	−.276 (−.641 to .090)	.14
	Introduction of solid foods before 4 month	1.225 (.429 to 2.021)	.003
	Food used as a reward at 12 month	.063 (−.180 to .307)	.61
	Drinking from a cup at 12 month	−.101 (−.341 to .138)	.41
	Taking a bottle to bed at 12 month	.239 (.002 to .477)	.048
	BF^e^ duration (month)	.001 (−.031 to .034)	.94
**BMI z-score at 24 months**			
	Household registration	−.110 (−.319 to .098)	.30
	Rental accommodation	−.114 (−.370 to .142)	.38
	Awareness of WHO breastfeeding guidelines at baseline	.010 (−.173 to .194)	.91
	Maternal pre-pregnancy BMI	.010 (−.026 to .046)	.58
	Maternal education	−.009 (−.221 to .203)	.93
	Birthweight	.458 (.252 to .664)	<.001
	Maternal age at recruitment	−.038 (−.197 to .121)	.64
	EBF at 6 months	−.230 (−.510 to .050)	.11
	Introduction of solid foods before 4 month	.888 (.278 to 1.498)	.004
	Food used as a reward at 12 month	.033 (−.153 to .219)	.73
	Drinking from a cup at 12 month	−.064 (−.247 to .120)	.495
	Taking a bottle to bed at 12 month	.187 (.005 to .369)	.04
	BF duration (month)	.003 (−.022 to .028)	.80
**Weight-for-length z-score at 24 months**			
	Household registration	−.113 (−.312 to .085)	.26
	Rental accommodation	−.119 (−.363 to .124)	.34
	Awareness of WHO breastfeeding guidelines at baseline	.012 (−.163 to .186)	.90
	Maternal pre-pregnancy BMI	.010 (−.024 to .044)	.56
	Maternal education	.004 (−.198 to .206)	.97
	Birthweight	.518 (.322 to .714)	<.001
	Maternal age at recruitment	−.029 (−.181 to .122)	.70
	EBF at 6 months	−.241 (−.507 to .025)	.08
	Introduction of solid foods before 4 month	.894 (.313 to 1.474)	.003
	Food used as a reward at 12 month	.036 (−.141 to .213)	.69
	Drinking from a cup at 12 month	−.057 (−.231 to .117)	.52
	Taking a bottle to bed at 12 month	.186 (.013 to .359)	.04
	BF duration (month)	−.002 (−.026 to .022)	.86

^a^Multiple linear regression: The number of participants with both infant feeding practice data at 12 months and BMI data at 24 months was 359 in total, 197 from the intervention group, and 162 from the control group.

^b^BMI: body mass index.

^c^WHO: World Health Organization.

^d^EBF: exclusive breastfeeding.

^e^BF: breastfeeding.

## Discussion

### Principal Findings

Our study found that the SMS intervention, delivered to mothers on a weekly basis from the third trimester until 12 months postpartum, had no significant effect on their children’s BMI, BMI z-score, or weight-for-length z-score at 12 and 24 months. Higher birth weight, introduction of solid foods before 4 months, and taking a bottle to bed at 12 months were found significantly and positively associated with higher BMI, BMI z-score, and weight-for-length z-score at 24 months.

### Strength and Limitations

The study was a quasi-experimental design, community-based intervention, with a high follow-up rate at 12 and 24 months. The SMS intervention was proven to be feasible and practical in improving breastfeeding practices, including significantly higher EBF rate at 6 months, longer median duration of EBF, and lower rate of instruction of solid foods before 4 months [15,17]. Although the intervention did not demonstrate significant effect on children’s BMI or BMI z-score at 24 months, several infant feeding practices were identified as potentially important influencing factors. There are several limitations of the study. Participants were not randomized to the intervention and control groups, and some characteristics, for example, maternal age, education, and birth weight, were significantly different between the 2 groups. Therefore, multiple regression was used to control confounding factors. Moreover, although SMS intervention is seen to be low cost, and potentially suitable for low resource settings, the cost-effectiveness of SMS intervention was not explored in our study. Information on other possible risk factors of obesity at 24 months, such as physical activity levels and TV viewing time, were not collected.

### Interpretation of the Research Findings

Among the published early childhood obesity prevention studies, the Healthy Beginnings Trial is the only study that showed a significant reduction in BMI (0.38 kg/m^2^ mean decrease, *P*=.01) in children of the intervention group at 24 months [8,21]. This trial was a multicomponent intervention delivered through 8 home visits by trained community nurses [8]. The NOURISH trial, comprising 2 interactive group education modules, each delivering over 3 months to new mothers to promote healthy eating patterns, demonstrated a small but not statistically significant reduction in mean BMI z-score of 0.14 (*P*=.12) [22]. Compared with these 2 studies, our intervention, with 1 weekly text message with no more than 210 characters, had much lower intensity. This could be one explanation for not showing effect on BMI z-score or the BMI. The results could also be due to a type 2 error-inadequate statistical power to detect the difference because there was a limited reference for postulating a meaningful expected difference for estimating sample size.

Whether early introduction of solid foods affects BMI in later childhood is still a contested topic. Some studies suggest that early introduction of solids is associated with increased childhood BMI [23], whereas others did not support such a conclusion [24,25]. We found that the introduction of solid foods before 4 months was positively associated with a higher BMI, BMI z-score, and weight-for-length z-score at 24 months. This finding is in keeping with previous reported studies [26,27]. No association between EBF or duration of breastfeeding with BMI, BMI z-score, and weight-for-length z-score at 24 months was found, which was consistent with 1 prospective study [28], but different to other studies’ findings [26,29]. Further research is needed to confirm breastfeeding promotion as a robust childhood obesity prevention strategy [30].

Our study showed that giving an infant a bottle to take to bed at 12 months, which is a less reported practice, was positively associated with a higher BMI, BMI z-score, and weight-for-length z-score at 24 months. However, we found no significant association between drinking from a cup at 12 months and anthropometric status at the age of 24 months. This is consistent with a randomized controlled trial that also failed to show an association between reduced bottle usage from 12 months and adiposity at 24 months [31]. However, links were found between bottle use at 24 months and higher obesity prevalence at the age of 5.5 years in an observational study [32].

### Future Research

mHealth intervention as an effective strategy to promote breastfeeding and infant feeding and childhood obesity prevention still requires further research using stronger study designs and larger sample sizes. Research on motivations and barriers for mothers to adopt healthy child feeding behaviors is needed to inform the development of effective interventions. The cost-effectiveness of SMS interventions compared with facility-based interventions also needs to be determined.

### Conclusions

Overweight and obesity in children are affected by many factors. Although no significant effect of this SMS intervention on children’s BMI, BMI z-score, or weight-for-length z-score at 12 and 24 months was found, we have identified several influencing factors. The introduction of solid foods before 4 months, taking a bottle to bed at 12 months, and high birth weight were significantly and positively correlated with a higher BMI, BMI z-score, and weight-for-length z-score at 24 months. Furthermore, large-scale randomized controlled trials are needed to evaluate the effectiveness and cost- effectiveness of SMS text messaging interventions on the prevention of early childhood obesity, including the usability, intensity, frequency, and duration of the interventions.

## Appendix

Multimedia Appendix 1 Characteristics of mothers and babies in the study, Shanghai China 2010-13.

Multimedia Appendix 2 Body mass index (BMI), BMI z-score and weight for length z-score (kg/m2) between children in the intervention and control groups at 12 and 24 months.

## Abbreviations

ANOVA: analysis of variance
BMI: body mass index
CHC: community health center
EBF: exclusive breastfeeding
mHealth: mobile health
SMS: short message service
WHO: World Health Organization
