# Authorship Correction: A Community-Based Short Message Service Intervention to Improve Mothers’ Feeding Practices for Obesity Prevention: Quasi-Experimental Study

**DOI:** 10.2196/15046

**Published:** 2019-07-18

**Authors:** Hong Jiang, Mu Li, Li Ming Wen, Louise Baur, Gengsheng He, Xiaoying Ma, Xu Qian

**Affiliations:** 1 School of Public Health Global Health Institute Fudan University Shanghai China; 2 Key Lab of Health Technology Assessment, National Health Commission of the People's Republic of China Fudan University Shanghai China; 3 School of Public Health University of Sydney Sydney Australia; 4 China Studies Centre University of Sydney Sydney Australia; 5 Health Promotion Unit Sydney Local Health District Sydney Australia; 6 Discipline of Child & Adolescent Health University of Sydney Sydney Australia

The authors of “A Community-Based Short Message Service Intervention to Improve Mothers’ Feeding Practices for Obesity Prevention: Quasi-Experimental Study” (JMIR Mhealth Uhealth 2019;7(6):e13828) wish to change the order of the authors on the publication so that Xu Qian is listed last.

The previous order of authorship was as follows:

Hong Jiang, Mu Li, Li Ming Wen, Louise Baur, Gengsheng He, Xu Qian, Xiaoying Ma

The correct order of authorship is as follows:

Hong Jiang, Mu Li, Li Ming Wen, Louise Baur, Gengsheng He, Xiaoying Ma, Xu Qian

Additionally, the affiliations listed for author Gengsheng He have also been updated. Previously, only affiliation 2 was listed for this author:

^2^Key Lab of Health Technology Assessment, National Health Commission of the People's Republic of China, Fudan University, Shanghai, China

The revised listing identifies that Gengsheng He is associated with affiliations 1 and 2:

^1^School of Public Health, Global Health Institute, Fudan University, Shanghai, China

^2^Key Lab of Health Technology Assessment, National Health Commission of the People's Republic of China, Fudan University, Shanghai, China

The corrections will appear in the online version of the paper on the JMIR website on July 18, 2019, together with the publication of this correction notice. Because this was made after submission to PubMed, PubMed Central, and other full-text repositories, the corrected article also has been resubmitted to those repositories.

