# Supplementary material for: A Community-Based Short Message Service Intervention to Improve Mothers’ Feeding Practices for Obesity Prevention: Quasi-Experimental Study
Source: JMIR Mhealth Uhealth. 2019 Jun 3;7(6):e13828. doi: 10.2196/13828 (PMC6638993; doi:10.2196/13828)
Supplement: Multimedia Appendix 1 [file mhealth_v7i6e13828_app1.docx]

Multimedia Appendix 1

Characteristics of mothers and babies in the study, Shanghai China 2010-13

|  |  | Baseline | | | 12 months | | | 24 months | | | |
| --- | --- | --- | --- | --- | --- | --- | --- | --- | --- | --- | --- |
|  |  | Intervention  n=281 | Control  n=301 | *P* value | Intervention  n=254 | Control  n=223 | *P* value | Intervention  n=243 | Control  n=224 | *P* value |  |
|  |  |  |  |  |  |  |  |  |  |  |  |
| Maternal age(years) | | | | | | | | | | | |
|  | <25 | 42(15.0) | 26(8.6) | .04^a^ | 33(13.0) | 18(8.1) | .13^a^ | 33(13.6) | 19(8.5) | .16^a^ |  |
|  | 25-29 | 164(58.4) | 182(60.4) |  | 151(59.4) | 136(61.0) |  | 142(58.4) | 131(58.5) |  |  |
|  | ≥30 | 75(26.69) | 93(30.9) |  | 70(27.6) | 69(30.9) |  | 68(28.0) | 74(33.0) |  |  |
| Household registration | | | | | | | | | | | |
|  | Non-Shanghai | 219 (77.9) | 221(73.4) | .21^a^ | 194(76.4) | 162(72.6) | .35^a^ | 184(75.7) | 159(71.0) | .25^a^ |  |
|  | Shanghai | 62(22.1) | 80(26.6) |  | 60(23.6) | 61(27.4) |  | 59(24.3) | 65(29.0) |  |  |
| Rental accommodation | | | | | | | | | | | |
|  | Yes | 78(27.8) | 57(18.9) | .01^a^ | 61(24.0) | 35(15.7) | .02^a^ | 57(23.45) | 32(14.3) | .01^a^ |  |
|  | No | 203(72.2) | 244(81.1) |  | 193(76.0) | 188(84.3) |  | 186(76.5) | 192(85.7) |  |  |
| Education level | | | | | | | | | | | |
|  | Junior middle school | 15(5.3) | 5(1.7) | <.001^b^ | 13(5.1) | 3(1.3) | .004^b^ | 13(5.3) | 3(1.3) | .004^b^ |  |
|  | Senior middle school | 39(13.9) | 20(6.6) |  | 29(11.4) | 16(7.2) |  | 28(11.5) | 13(5.8) |  |  |
|  | College and above | 227(80.8) | 276(91.7) |  | 212(83.5) | 204(91.5) |  | 202(83.1) | 208(92.9%) |  |  |
| Awareness of WHO breastfeeding guidelines at baseline | | | | | | | | | | | |
|  | High | 124(44.1) | 198(65.8) | <.001^a^ | 110(43.3) | 143(64.1) | <.001^a^ | 105(43.2) | 145(64.7) | <.001^a^ |  |
|  | Low | 157(55.9) | 103(34.2) |  | 144(56.7) | 80(35.9) |  | 138(56.8) | 79(35.3) |  |  |
| Baby’s sex | | | | | | | | | | | |
|  | Girl | 139(49.5) | 161(53.5) | .33^a^ | 125(49.2) | 121(54.3) | .27^a^ | 117(48.1) | 120(53.6) | .24^a^ |  |
|  | Boy | 142(50.5) | 140(46.5) |  | 129(50.8) | 102(45.7) |  | 126(51.9) | 104(46.4) |  |  |
| Birthweight (kg, mean±SD) | | | | | | | | | | | |
|  |  |  |  |  | 3.34±0.46 | 3.42±0.41 | .04^c^ | 3.34±0.46 | 3.42±0.43 | .05^c^ |  |

^a^ Pearson’s Chi-Squared test

^b^ Mantel-Haenszel chi-squared test

^c^ t-test
