# Supplementary material for: A Community-Based Short Message Service Intervention to Improve Mothers’ Feeding Practices for Obesity Prevention: Quasi-Experimental Study
Source: JMIR Mhealth Uhealth. 2019 Jun 3;7(6):e13828. doi: 10.2196/13828 (PMC6638993; doi:10.2196/13828)
Supplement: Multimedia Appendix 2 [file mhealth_v7i6e13828_app2.docx]

Multimedia Appendix 2

BMI, BMI z-score and weight for length z-score (kg/m^2^) between children in the intervention and control groups at 12 and 24 months

|  | Unadjusted^a^ | | | Adjusted^b^ | | | | |
| --- | --- | --- | --- | --- | --- | --- | --- | --- |
|  | Intervention group | Control group | Mean difference (95%CI)  Intervention-Control | Intervention group | Control group | Adjusted Beat | 95%CI for Beta | *P* value |
|  | Mean (SE) | Mean (SE) |  | Mean (SE) | Mean (SE) |  |  |  |
| At 12 months | n=254 | n=223 |  | n=254 | n=223 |  |  |  |
| Length (cm) | 76.84 (0.16) | 77.22 (0.16) | -0.39 (-0.83, 0.06) | 76.93(0.14) | 77.12(0.15) | -.19 | (-.59 to .21) | .35 |
| Weight (kg) | 10.06 (0.06) | 10.19 (0.07) | -0.12 (-0.30,0.06) | 10.10(0.06) | 10.15(0.06) | -.05 | (-.22 to .11) | .53 |
| BMI (kg/m^2^) | 17.02 (0.07) | 17.06(0.09) | -0.04 (-0.26, 0.18) | 17.04(0.08) | 17.04 (0.08) | .01 | (-.22 to .23) | .97 |
| BMI z-score | 0.45 (0.05) | 0.48 (0.06) | -0.03(-0.18, 0.12) | 0.47(0.05) | 0.46(0.06) | .01 | (-.14 to .16) | .90 |
| Weight for length z-score | 0.41(0.05) | 0.47(0.06) | -0.06(-0.20, 0.09) | 0.44(0.05) | 0.44(0.05) | -.01 | (-.15 to .14) | .95 |
| At 24 months | n=243 | n=224 |  | n=243 | n=224 |  |  |  |
| Length(cm) | 89.49(1.96) | 90.01(1.90) | -0.52(-1.06,0.02) | 89.60(0.17) | 89.90(0.17) | -.29 | (-.77 to .19) | .23 |
| Weight (kg) | 12.78(0.09) | 12.90(0.08) | -0.12(-0.36,0.11) | 12.80(0.08) | 12.87(0.08) | -.07 | (-.29 to .16) | .56 |
| BMI (kg/m^2^) | 15.93(0.07) | 15.91(0.08) | 0.02(-0. 19, 0.23) | 15.93(0.07) | 15.90(0.08) | .03 | (-.18 to .24) | .78 |
| BMI z-score | 0.05(0.06) | 0.05(0.06) | 0.01(-0.16, 0.17) | 0.06(0.06) | 0.04(0.06) | .02 | (-.15 to .18) | .86 |
| Weight for length z-score | 0.23(0.05) | 0.24(0.06) | -0.01(-0.17, 0.14) | 0.24(0.05) | 0.23(0.06) | .01 | (-.15 to .16) | .91 |

^a^ Independent- Samples T-test;

^b^ ANOVA: For length at 12 and 24 months, adjusted for birth weight, mothers’ age, mothers’ education level, maternal household registration, whether in rental accommodation, baseline WHO breastfeeding guideline awareness, baby’s sex, month of physical check-up, mothers’ pre-conception height;

For weight at 12 and 24 months, adjusted for birth weight, mothers’ age, mothers’ education level, maternal household registration, whether in rental accommodation, baseline WHO breastfeeding guideline awareness, baby’s sex, month of physical check-up, mothers’ pre-conception weight;

For BMI at 12 and 24 months, adjusted for birth weight, mothers’ age, mothers’ education level, maternal household registration, whether in rental accommodation, baseline WHO breastfeeding guideline awareness, baby’s sex, month of physical check-up, mothers’ pre-conception BMI;

For BMI z-score and weight for length z-score at 12 and 24 months, adjusted for birth weight, mothers’ age, mothers’ education level, mother’s household registration, whether in rental accommodation, baseline WHO breastfeeding guideline awareness, mothers’ pre-conception BMI.
